# Risk factors, transcriptomics, and outcomes of myocardial injury following lower extremity revascularization

**DOI:** 10.1038/s41598-022-10241-9

**Published:** 2022-04-25

**Authors:** Nathaniel R. Smilowitz, MacIntosh Cornwell, Erik J. Offerman, Caron B. Rockman, Svati H. Shah, Jonathan D. Newman, Kelly Ruggles, Deepak Voora, Jeffrey S. Berger

**Affiliations:** 1 Leon H. Charney Division of Cardiology, Department of Medicine, New York University School of Medicine, New York, NY USA; 2 Cardiology Section, Department of Medicine, Veterans Affairs New York Harbor Health Care System, New York, NY USA; 3 Institute for Systems Genetics, New York University School of Medicine, New York, NY USA; 4 Division of Translational Medicine, Department of Medicine, New York University School of Medicine, New York, NY USA; 5 Department of Surgery, New York University School of Medicine, New York, NY USA; 6 Duke Molecular Physiology Institute, Duke University School of Medicine, Durham, NC USA; 7 Center for Applied Genomics & Precision Medicine, Duke University School of Medicine, Durham, NC USA; 8 Center for the Prevention of Cardiovascular Disease, New York University School of Medicine, 530 First Avenue, Skirball 9R, New York, NY 10016 USA

**Keywords:** Gene expression, Genetics, Biomarkers, Cardiology, Diseases, Medical research

## Abstract

Myocardial injury after non-cardiac surgery (MINS) is common. We investigated the incidence and outcomes of MINS, and mechanistic underpinnings using pre-operative whole blood gene expression profiling in a prospective cohort study of individuals undergoing lower extremity revascularization (LER) for peripheral artery disease (PAD). Major adverse cardiovascular and limb events (MACLE) were defined as a composite of death, myocardial infarction, stroke, major lower extremity amputation or reoperation. Among 226 participants undergoing LER, MINS occurred in 53 (23.5%). Patients with MINS had a greater incidence of major adverse cardiovascular events (49.1% vs. 22.0%, adjusted HR 1.87, 95% CI 1.07–3.26) and MACLE (67.9% vs. 44.5%; adjusted HR 1.66, 95% CI 1.08–2.55) at median 20-month follow-up. Pre-operative whole blood transcriptome profiling of a nested matched MINS case–control cohort (n = 41) identified upregulation of pathways related to platelet alpha granules and coagulation in patients who subsequently developed MINS. Thrombospondin 1 (*THBS1*) mRNA expression was 60% higher at baseline in patients who later developed MINS, and was independently associated with long-term cardiovascular events in the Duke Catheterization Genetics biorepository cohort. In conclusion, pre-operative THBS1 mRNA expression is higher in patients who subsequently develop MINS and is associated with incident cardiovascular events. Pathways related to platelet activity and coagulation associated with MINS provide novel insights into mechanisms of myocardial injury.

## Introduction

Greater than 300 million surgeries are performed annually^1^. Perioperative cardiovascular events are feared complications of non-cardiac surgery^2^. In a retrospective study of surgical hospitalizations in the United States, perioperative death, myocardial infarction (MI), or ischemic stroke occurred in 3% of non-cardiac surgeries^3^. However, ischemic symptoms may be masked by post-operative anesthesia and analgesia, and many cardiovascular complications may go unrecognized^4^. Myocardial injury after non-cardiac surgery (MINS), with or without evidence of MI, is a common syndrome that is characterized by ≥ 1 troponin concentrations that exceed the 99th percentile upper reference limit of the assay, detected in 16–20% of patients after major operative procedures, and independently associated with short-term mortality^4–6^. Pathophysiological mechanisms of MINS are currently unknown^4,7^.

Vascular surgery is associated with excess perioperative cardiovascular risk and a high incidence of MINS^3,8^. Lower extremity revascularization is frequently performed to improve morbidity in patients with symptomatic peripheral artery disease (PAD). Unfortunately, clinical predictors of MINS and long-term outcomes of MINS after lower extremity revascularization remain incompletely characterized. Further, predictive pre-operative biomarkers to inform post-operative risk stratification remain elusive. A circulating whole blood gene expression signature has been evaluated in several clinical settings, including cardiac allograft rejection, cardiovascular events, and coronary artery disease^9–12^. However, pre-operative whole blood gene expression data have not previously been used to evaluate mechanisms of MINS. To address this gap, we performed an analysis of a prospective cohort study of individuals with PAD undergoing lower extremity revascularization to investigate associations between clinical characteristics, whole blood gene expression profiles prior to revascularization, and the subsequent development of MINS. We also explored the relationship between pre-operative gene expression, MINS after lower extremity revascularization, and long-term outcomes.

## Methods

### Participants

Adults ≥ 21 years of age with severe PAD who were referred for non-emergent lower extremity revascularization at NYU Langone Health, Bellevue Hospital Center, and the Veterans Affairs New York Harbor Health Care System between March 2014 and November 2017 were eligible for participation in the Platelet Activity & Cardiovascular Events Following Vascular Surgery (PACE) study if they had severe PAD, defined as lower extremity ulceration, gangrene, pain at rest, or a resting ankle brachial index of < 0.6. Patients with non-steroidal anti-inflammatory drug use within 72 h of the procedure, thrombocytopenia or thrombocytosis, severe anemia (hemoglobin < 8 g/dL), or known history of hemorrhagic diathesis were excluded^13,14^.

### Ethics declarations

The study was approved by the New York University Grossman School of Medicine Institutional Review Board and was performed in accordance with relevant institutional guidelines and regulations. Informed consent was obtained from all study participants. The study was funded by the National Heart, Lung, And Blood Institute of the National Institutes of Health.

### Study procedures

Peripheral venous blood was collected from all participants for routine laboratory testing immediately prior to LER. Peripheral whole blood RNA was collected into PAXgene Blood RNA tubes (Becton Dickinson and Company, Franklin Lakes, New Jersey) and immediately processed and frozen at − 80 °C. For this study, RNA extraction was performed in these stored samples. Lower extremity revascularization was performed according to usual clinical care. Cardiac troponin I was measured pre-operatively, on post-operative day 2 (± 1 day), and at other time points when clinically indicated, using the VITROS cardiac Troponin I ES assay (Ortho Clinical Diagnostics, Rochester, NY; 99th percentile ULN > 0.04 ng/mL), or the ST AIA-PACK 2nd generation cardiac troponin I assay (Tosoh Bioscience, Tokyo, Japan; 99th percentile ULN > 0.06 ng/mL). High sensitivity troponin assays (hs-Tn) were not used. Assay-specific troponin thresholds were used for all analyses.

### Myocardial injury after non-cardiac surgery (MINS)

Myocardial injury after non-cardiac surgery was defined as an elevated post-operative cardiac troponin (cTn) within first 72 h following surgery, with at least one cTn measurement above the 99th percentile upper limit of normal (ULN) for the cTn assay (0.04 ng/dL or 0.06 ng/dL). As clinical symptoms may be masked by sedation or analgesia in the perioperative setting, an ischemic feature (e.g. ischemic symptoms, electrocardiographic changes) was not required for a diagnosis of MINS, as previously defined^7,15^. Among participants with abnormal baseline troponin values, myocardial injury was considered to be acute if there was a ≥ 20% rise of cTn after non-cardiac surgery. Participants who did not have pre-operative troponin measured but had a post-operative cTn > 99th percentile ULN were assigned a presumptive diagnosis of MINS.

### Post-operative clinical outcomes

All participants were followed for at least 30 days following revascularization, with subsequent follow up by telephone or during routine clinical visits at 6 months and then every 6 months thereafter. Major adverse cardiovascular events (MACE) were defined as the composite of death, myocardial infarction, or stroke. Major adverse limb events (MALE) were defined as lower extremity major amputation or reintervention^16^. Major adverse cardiovascular and limb events (MACLE) were defined as the composite of death, myocardial infarction, stroke, lower extremity major amputation, or major reintervention. Two cardiologists and a vascular surgeon blinded to all transcriptome analyses adjudicated all events by review of medical records.

### Transcriptome profiling

Transcriptome profiling was performed as previously described^17^. Automated RNA extraction from Stored PAXgene Blood RNA tubes was performed using the QIAsymphony PAXgene Blood RNA Kit (PreAnalytiX, Qiagen/BD). Prior to RNA sequencing, yield, quantity, and quality of the RNA were assessed using an Illumina HiSeq 4000 v4 chemistry single read (Illumina, Inc, San Diego, CA). RNA sequencing libraries were generated with the Illumina TruSeq Library kit (San Diego, CA) and 200 ng total RNA were used as starting input per sample. Samples underwent 12 cycles of amplification. Completed libraries were quantitated, normalized, and pooled.

RNA sequencing data were analyzed in a nested, age- and sex- matched group of MINS cases (N = 22) and no-MINS controls (N = 22). FASTQ files from RNA-sequencing were processed using the Seq-N-Slide pipeline^18^. Reads were trimmed using trimmomatic v0.36, aligned to the hg38 genome using STAR v2.6.1, and quantified using featureCounts v1.6*.*3^19–21^. Read quality was assessed using FASTQC v0.11.7 and fastqscreen v0.13^22,23^. Three samples were omitted due to low quality of data and aberrant expression patterns. Samples had an average read depth 17,679,206. Gene transcripts were filtered below a determined background level to identify expressed genes. As expected, the distribution for expression of each transcript was bimodal, and we determined a cut of 4 log2 normalized expression in at least half of samples resulting in a filtering of 57,316 total genes to 13,211 genes for downstream analysis.

All downstream analysis was performed in R v3.6.1 (R Foundation for Statistical Computing, Vienna, Austria). Differential expression analysis was performed via DESeq2 v1.24.0 using MINS case–control status as the separating variable and age and sex as covariates to determine genes of interest associated with MINS. Multiple hypothesis correction was done using the Benjamini–Hochberg method. Gene Set Enrichment Analysis (GSEA) was performed on all genes using clusterProfiler v3.12.0^24^, enriching for genesets provided via msigdbr v7.0.1^25^. Heatmaps were created using ComplexHeatmap v2.0.0 and all plotting was done using ggplot2 v3.2.1^26^.

### Gene expression and long-term cardiovascular outcomes

To explore the association between expression of genes associated with MINS and long-term cardiovascular outcomes, we analyzed longitudinal follow-up data from the PACE and CATHGEN studies. In PACE, we evaluated associations between genes of interest and long-term survival in all 106 individuals who had had RNASeq performed on peripheral blood. Cox proportional hazard models were generated to estimate relationships between tertiles of expression and mortality after adjustment for age and sex. Kaplan–Meier curves were used to illustrate long-term outcomes of participants by tertile of gene expression and were compared with a log-rank test.

Association between gene expression and long-term outcomes was also performed in the Duke Catheterization Genetics (CATHGEN) biorepository, in observational (N = 190) and case–control (N = 397) cohorts, using existing data generated as previously described^12^. The CATHGEN study collected arterial whole blood in PAXgene Blood RNA tubes from participants undergoing coronary angiography for suspected ischemic heart disease; median longitudinal follow-up at the time of analysis was 3.8 years. Briefly, two CATHGEN cohorts with available data were pooled for this analysis: (a) an unselected cohort of patients (N = 190) and (b) a case–control cohort of patients with incident death/MI who were matched by age, sex, and race to event-free controls > 2 years after catheterization (N = 397). Gene expression was previously evaluated using Affymetrix U133 plus 2.0 microarrays with Robust Multichip Average (RMA) method used for normalization. Associations between the probe sets mapping to the genes of interest and all-cause death or MI during follow-up were evaluated using (a) empirical Bayes linear regression models adjusted for age, sex, and race to generate log2 fold changes and (b) logistic regression models controlling for age, sex, and race to generate log odds ratios^12^. Fixed and random effects meta-analysis methods were used to combine evidence of differential expression from the two CATHGEN cohorts, with pooled weighted average of log2 fold changes or log odds ratios derived using inverse variance weights. All CATHGEN data processing was conducted using R packages affy, limma, and meta for normalization, moderated t-tests, and meta-analyses, respectively.

### Statistical analysis

Continuous variables with a normal distribution are shown as mean and standard deviation and were compared using independent samples T-tests. Non-normally distributed continuous data are shown as median and interquartile range (IQRs) and were compared using Mann–Whitney U-tests. Categorical variables were described as counts and percentages, and compared using Chi-square or Fisher’s exact tests. To explore predictors of MINS, multivariable logistic regression models were generated to estimate the associations between MINS and clinical covariates, age, sex, race, ethnicity, pre-operative creatinine, and all other baseline pre-operative covariates with fewer than 5 missing values and with univariate p-values < 0.1. Candidate variables included body mass index (BMI), tobacco use, hypertension, hyperlipidemia, diabetes mellitus, prior myocardial infarction, history of transient ischemic attack or stroke, heart failure, venous thromboembolism, chronic obstructive pulmonary disease, obstructive sleep apnea, malignancy, and laboratory parameters, including hemoglobin and lipid levels.

Kaplan–Meier curves were generated to illustrate long-term outcomes in participants with and without MINS and compared with a log-rank test. Cox proportional hazard models were generated to estimate the relationship between MINS and long-term outcomes before and after adjustment for baseline demographics, coronary artery disease, heart failure, baseline serum creatinine, and the surgical approach to revascularization.

Sensitivity analyses were performed excluding participants with presumed diagnoses of MINS in whom pre-operative baseline troponins were not available, and individuals who had abnormal cardiac biomarkers post-operatively but who did not meet a threshold of a 20% rise/fall necessary for a diagnosis of MINS.

A two-tailed p-value < 0.05 was considered statistically significant. P-values in both the cohort level and meta-analyses of CATHGEN data were controlled for multiple testing using the Benjamini-Hochburg correction. All statistical analyses of perioperative data were conducted using R v3.6.1 (R Foundation for Statistical Computing, Vienna, Austria) and SPSS 25 (IBM SPSS Statistics, Armonk, NY).

## Results

### Participants

A total of 289 participants with severe PAD were enrolled in the PACE study and underwent a lower extremity revascularization procedure. Of these, 63 (21.8%) did not have post-operative troponin measured. The remaining 226 participants were included in this analysis. Characteristics of patients with and without post-operative troponin measurement are shown in Supplemental Table 1. Ninety participants (39.8%) had a single post-operative troponin measurement, 136 (60.2%) had ≥ 2 measurements, and 117 (51.8%) had ≥ 3 measurements. Pre-operative troponin values were measured in 143 (63.3%) of eligible participants. Demographic and clinical characteristics are described in Table 1. The mean age was 72.7 ± 10.8 years, 35% were female, 62% were white. Cardiovascular risk factors and clinical comorbidities were common. Open surgical bypass (n = 114), endovascular revascularization (n = 77), and hybrid (n = 25) procedures were performed.

**Table 1:** Characteristics of patients with MINS and without MINS.

	All	No MINS	MINS	p-value
	(n = 226)	(n = 173)	(n = 53)	
Age, median [IQR]	73 [66.0–81.0]	73.0 [66.0–80.0]	74.0 [68.0–82.0]	0.41
Female sex	79 (35.0%)	59 (34.1%)	20 (37.7%)	0.75
**Race**				0.38
White	141 (62.4%)	104 (60.1%)	37 (69.8%)	
African American	48 (21.2%)	39 (22.5%)	9 (17.0%)	
Asian	5 (2.2%)	3 (1.7%)	2 (3.8%)	
Other	32 (14.2%)	27 (15.6%)	5 (9.4%)	
**Ethnicity**				
Hispanic/Latino	40 (17.7%)	35 (20.2%)	5 (9.4%)	0.11
BMI, median [IQR]	26.1 [23.0–29.7]	26.6 [23.5–29.7]	24.5 [22.4–28.9]	0.08
**Smoking Status**				0.83
Current	37 (16.4%)	29 (16.8%)	8 (15.1%)	
Former	132 (58.4%)	102 (59.0%)	30 (56.6%)	
Never	57 (25.2%)	42 (24.3%)	15 (28.3%)	
**Comorbidities**				
Hypertension	202 (89.4%)	158 (91.3%)	44 (83.0%)	0.14
Hyperlipidemia	163 (72.1%)	123 (71.1%)	40 (75.5%)	0.66
Diabetes mellitus	118 (52.2%)	85 (49.1%)	33 (62.3%)	0.13
Coronary artery disease	122 (54%)	81 (46.8%)	41 (77.4%)	0.0002
Prior myocardial infarction	64 (28.3%)	39 (22.5%)	25 (47.2%)	0.0009
Heart failure	47 (20.8%)	29 (16.8%)	18 (34.0%)	0.01
Prior stroke/TIA	41 (18.1%)	29 (16.8%)	12 (22.6%)	0.44
Obstructive sleep apnea	8 (3.5%)	8 (4.6%)	0 (0%)	0.24
Chronic obstructive pulmonary disease	39 (17.3%)	31 (17.9%)	8 (15.1%)	0.79
Malignancy	51 (22.6%)	40 (23.1%)	11 (20.8%)	0.86
**Pre-operative medications**				
ACEi/ARB	122 (54.0%)	93 (53.8%)	29 (54.7%)	0.99
Beta-blockers	137 (60.6%)	100 (57.8%)	37 (69.8%)	0.16
Statin	185 (81.9%)	173 (80.3%)	46 (86.8%)	0.40
**Laboratory data**^**a**^				
Pre-operative troponin (ng/mL), median [IQR]	0.012 [0.012–0.026]	0.012 [0.012–0.014]	0.028 [0.012–0.05]	< 0.001
Cr (mg/dL), median [IQR]	1.00 [0.80–1.30]	1.00 [0.800–1.30]	1.10 [0.800–1.60]	0.26
Hgb (g/dL), median [IQR]	12.1 [10.4–13.5]	12.3 [10.5–13.7]	11.7 [10.0–13.2]	0.09
HbA1c (%),median [IQR]	6.5 [5.8–7.7]	6.35 [5.83–7.60]	7.05 [5.80–8.00]	0.41
LDL (mg/dL), median [IQR]	64.0 [50.0–91.0]	61.5 [49.5–87.5]	65.5 [55.0–97.8]	0.52
HDL (mg/dL), median [IQR]	40.0 [32.0–50.0]	39.0 [32.0–47.0]	40.0 [31.0–50.0]	0.68

^a^Laboratory data available in a subset of participants: Pre-operative troponin n = 143 (No MINS n = 106, MINS: n = 37). Creatinine n = 224. Hgb n = 226. HbA1c n = 124. LDL n = 84. HDL n = 86.

### Myocardial injury after non-cardiac surgery

Myocardial injury after non-cardiac surgery (MINS) occurred in 53 participants (23.5%), with a median peak troponin of 0.137 ng/mL (IQR 0.067–0.748; range 0.043–14.3). The incidence of MINS was 23.7% following open surgical revascularization, 19.5% after endovascular procedures, and 36.0% after hybrid procedures. Baseline demographics and clinical characteristics of patients with and without MINS are shown in Table 1. There was no difference in age, sex or race between groups. Participants with MINS were more likely to have a history of coronary artery disease, prior myocardial infarction, and heart failure than those without MINS. There was no difference in the use of beta-blockers or angiotensin-converting enzyme inhibitors (ACEi)/angiotensin receptor blockers (ARB) in patients with and without MINS (Table 1). Among 143 participants with a pre-operative troponin measurement, 27 (18.9%) had a troponin concentration above the ULN. Participants with MINS had higher peak pre-operative troponins than those without MINS (0.028 ng/mL [0.012–0.05] vs. 0.012 [0.012–0.014], p < 0.001).

Procedural characteristics of patients with and without MINS are shown in Table 2. Participants who developed MINS had longer surgical procedures, lower intra-operative diastolic blood pressures, lower post-operative hemoglobin values, and higher post-operative serum creatinine. Characteristics that were independently associated with MINS are shown in Supplemental Table 2. After adjustment for demographics and clinical covariates, only a pre-operative diagnosis of coronary artery disease was independently associated with MINS after lower extremity revascularization.

**Table 2:** Surgical characteristics of lower extremity revascularization.

	All	No MINS	MINS	p-value
	(n = 226)	(n = 173)	(n = 53)	
**Peripheral artery disease classification**				0.34
Stable peripheral artery disease	36 (15.9%)	30 (17.3%)	6 (11.3%)	
Critical limb ischemia	187 (82.7%)	140 (80.9%)	47 (88.7%)	
Femoral or popliteal aneurysm	3 (1.3%)	3 (1.7%)	0 (0%)	
**Disease presentation**				
ABI < 0.60	121 (53.5%)	94 (54.3%)	27 (50.9%)	0.78
Claudication	94 (41.6%)	81 (46.8%)	13 (24.5%)	0.006
Rest pain	94 (41.6%)	74 (42.8%)	20 (37.7%)	0.62
Ulceration	98 (43.4%)	68 (39.3%)	30 (56.6%)	0.039
Gangrene	58 (25.7%)	39 (22.5%)	19 (35.8%)	0.078
**Approach**				0.32
Open	114 (50.4%)	87 (50.3%)	27 (50.9%)	
Endovascular (with Intervention)	77 (34.1%)	61 (35.3%)	16 (30.2%)	
Hybrid	25 (11.1%)	16 (9.2%)	9 (17.0%)	
Endovascular (no intervention)	10 (4.4%)	9 (5.2%)	1 (1.9%)	
Operation time (h), median [IQR]	3.19 [2.17–4.72]	3.00 [2.07–4.41]	4.27 [2.52–5.42]	0.009
**Intraoperative hemodynamics, median [IQR]**^**a**^				
Highest intra-op SBP (mmHg)	175 [152–199]	174 [153–198]	175 [149–200]	0.99
Highest intra-op DBP (mmHg)	81 [71–95]	82 [72–98]	79 [67–90]	0.12
Nadir intra-op SBP (mmHg)	90 [80–98]	89 [80–98]	90 [80–98]	0.94
Nadir intra-op DBP (mmHg)	40 [32–50]	40 [33–50]	39 [22–45]	0.04
**Blood loss and anemia, median [IQR]**^**a**^				
Estimated blood loss (ml)	125 [50–300]	100 [50–250]	200 [75–500]	0.03
Post-op nadir hemoglobin (mg/dL)	8.9 [7.7–10.6]	9.2 [8.0–10.9]	7.9 [7.2–9.7]	< 0.001
Post-op creatinine (mg/dL)	1.0 [0.8–1.5]	1.0 [0.80–1.3]	1.3 [0.80–2.3]	0.006

*DBP* diastolic blood pressure, *SBP* systolic blood pressure.

^a^Intraoperative hemodynamic and laboratory data were available in a subset of patients.

### Medical management of MINS at discharge and at 30-days post-operatively

Among patients who survived to hospital discharge after lower extremity revascularization, 93.7% of patients received antiplatelet therapy, 38.7% received anticoagulation, and 84.2% received statin therapy. Dual antiplatelet therapy was used in 36.9% of individuals. There were no differences in prescribing in patients with versus without MINS. Medical therapy prescribed at discharge is shown in Table 3.

**Table 3:** Medical therapy at discharge in patients undergoing lower extremity revascularization with and without MINS.

	Overall	No MINS	MINS	p-value
	(n = 222)	(n = 172)	(n = 50)	
**Antiplatelet**				
Any antiplatelet	208 (93.7%)	159 (92.4%)	49 (98.0%)	0.20
Aspirin	171 (77.0%)	131 (76.2%)	40 (80.0%)	0.71
Clopidogrel	119 (53.6%)	88 (51.2%)	31 (62.0%)	0.23
Dual antiplatelet	82 (36.9%)	60 (34.9%)	22 (44.0%)	0.31
No antiplatelet	14 (6.3%)	13 (7.6%)	1 (2.0%)	0.20
**Anticoagulation**				
Any anticoagulation	86 (38.7%)	64 (37.2%)	22 (44.0%)	0.48
Direct oral anticoagulant	35 (15.8%)	24 (14.0%)	11 (22.0%)	0.25
Warfarin	36 (16.2%)	30 (17.4%)	6 (12.0%)	0.48
Low molecular weight heparin	15 (6.8%)	14 (8.1%)	1 (2.0%)	0.20
No anticoagulation	136 (61.3%)	108 (62.8%)	28 (56.0%)	0.48
Statin	187 (84.2%)	143 (83.1%)	44 (88.0%)	0.54
Cilostazol	18 (8.1%)	16 (9.3%)	2 (4.0%)	0.36

Excludes patients who died or were discharged to hospice.

### Clinical outcomes of MINS

**Figure 1:**
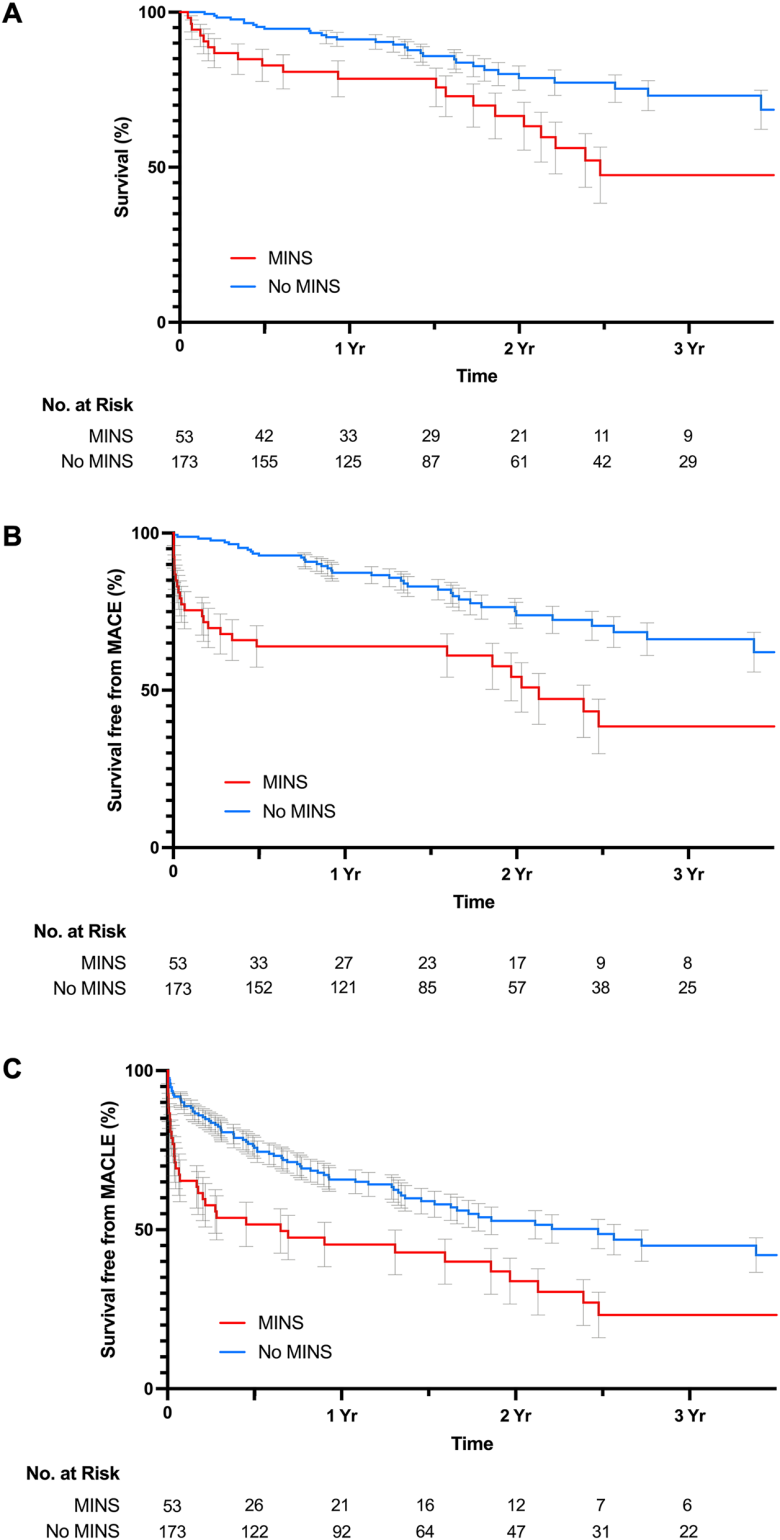
(**A**) Freedom from mortality after lower extremity revascularization. Log rank p-value = 0.003. Hazard Ratio 2.31 (95% CI 1.31–4.07). (**B**) Freedom from the composite of death, myocardial infarction, or stroke after lower extremity revascularization. Log rank p-value <0.0001. Hazard Ratio 2.74 (95% CI 1.66–4.52) (**C**) Freedom from the composite of death, myocardial infarction, stroke, lower extremity amputation or reintervention after lower extremity revascularization. Log rank p-value <0.001.  Hazard ratio 1.99 (CI 1.34–2.95).

Participants were followed post-operatively for a median of 20 months (IQR 10–29 months). Among 226 participants who had troponin levels measured, 50 participants died (22.1%), 64 had MACE (28.3%), and 113 had MACLE (50.0%). Patients with MINS were significantly more likely to have cardiovascular events post-operatively (Table 4). Kaplan Meier curves illustrate survival (Fig. 1A), freedom from MACE (Fig. 1B), and MACLE (Fig. 1C) after lower extremity revascularization. A diagnosis of MINS was associated with unadjusted hazard for mortality (HR 2.31, 95% CI 1.31 – 4.07), MACE (HR 2.74, 95% CI 1.66–4.52), and MACLE (HR 1.99, 95% CI 1.34–2.95). In a multivariable model, MINS was independently associated with the endpoint of cardiac and limb events (MACLE; aHR 1.66, 95% CI 1.08–2.55) and MACE (aHR 1.87, 95% CI 1.07–3.25) but not mortality (aHR 1.42, 95% CI 0.76–2.67).

**Table 4:** Clinical outcomes over long-term follow up after lower extremity revascularization.

	All	No MINS	MINS	Unadjusted HR (95% CI)	Adjusted HR^a^ (95% CI)
	(n = 226)	(n = 173)	(n = 53)		
MACLE	113 (50.0%)	77 (44.5%)	36 (67.9%)	1.99 (1.34–2.95)	1.66 (1.08–2.55)
MACE	64 (28.3%)	38 (22.0%)	26 (49.1%)	2.74 (1.66–4.52)	1.87 (1.07–3.26)
MALE	71 (31.4%)	54 (31.2%)	17 (32.1%)	1.13 (0.66–1.95)	1.13 (0.63–2.02)
Death	50 (22.1%)	30 (17.3%)	20 (37.7%)	2.31 (1.31–4.07)	1.42 (0.76–2.67)
Myocardial infarction	25 (11.1%)	10 (5.8%)	15 (28.3%)	5.89 (2.64–13.12)	6.14 (2.50–15.11)
Death or MI	62 (27.4%)	36 (20.8%)	26 (49.1%)	2.98 (1.80–4.94)	2.07 (1.17–3.64)
Stroke	9 (4.0%)	7 (4.0%)	2 (3.8%)	1.01 (0.21–4.87)	0.57 (0.10–3.11)
Any amputation (major or minor)	64 (28.3%)	41 (23.7%)	23 (43.4%)	2.06 (1.23–3.44)	2.15 (1.22–3.77)
Death or any amputation	97 (42.9%)	63 (36.4%)	34 (64.2%)	2.12 (1.39–3.22)	1.90 (1.20–3.01)

*MACE* major adverse cardiovascular events, *MALE* major adverse limb events, *MACLE* major adverse cardiovascular and limb events.

^a^Cox proportional hazard models adjusted for age, sex, race/ethnicity, coronary artery disease, heart failure, baseline serum creatinine, and the surgical approach to revascularization.

In a sensitivity analysis excluding participants with presumed MINS who did not have a pre-operative troponin measured and excluding individuals with abnormal post-operative troponins that did not exhibit a ≥ 20% rise/fall pattern, similar associations between MINS and cardiovascular outcomes were observed (Supplemental Table 3).

### Blood transcriptome profiles associated with MINS

Whole blood RNA sequencing was performed in 109 subjects from this cohort, of which 82 had post-operative troponins measured, and 22 had MINS. A total of 20 participants with MINS (mean age 73.4 years, 30% female, peak post-operative troponin mean 1.13 ± 2.98 ng/mL, median 0.151 ng/mL [0.069–0.676]) were matched by age and sex to 21 participants without MINS (72.1 years, 38.1% female, peak post-operative troponin mean 0.021 ± 0.011 ng/mL, median 0.015 [0.012–0.025]).

**Figure 2:**
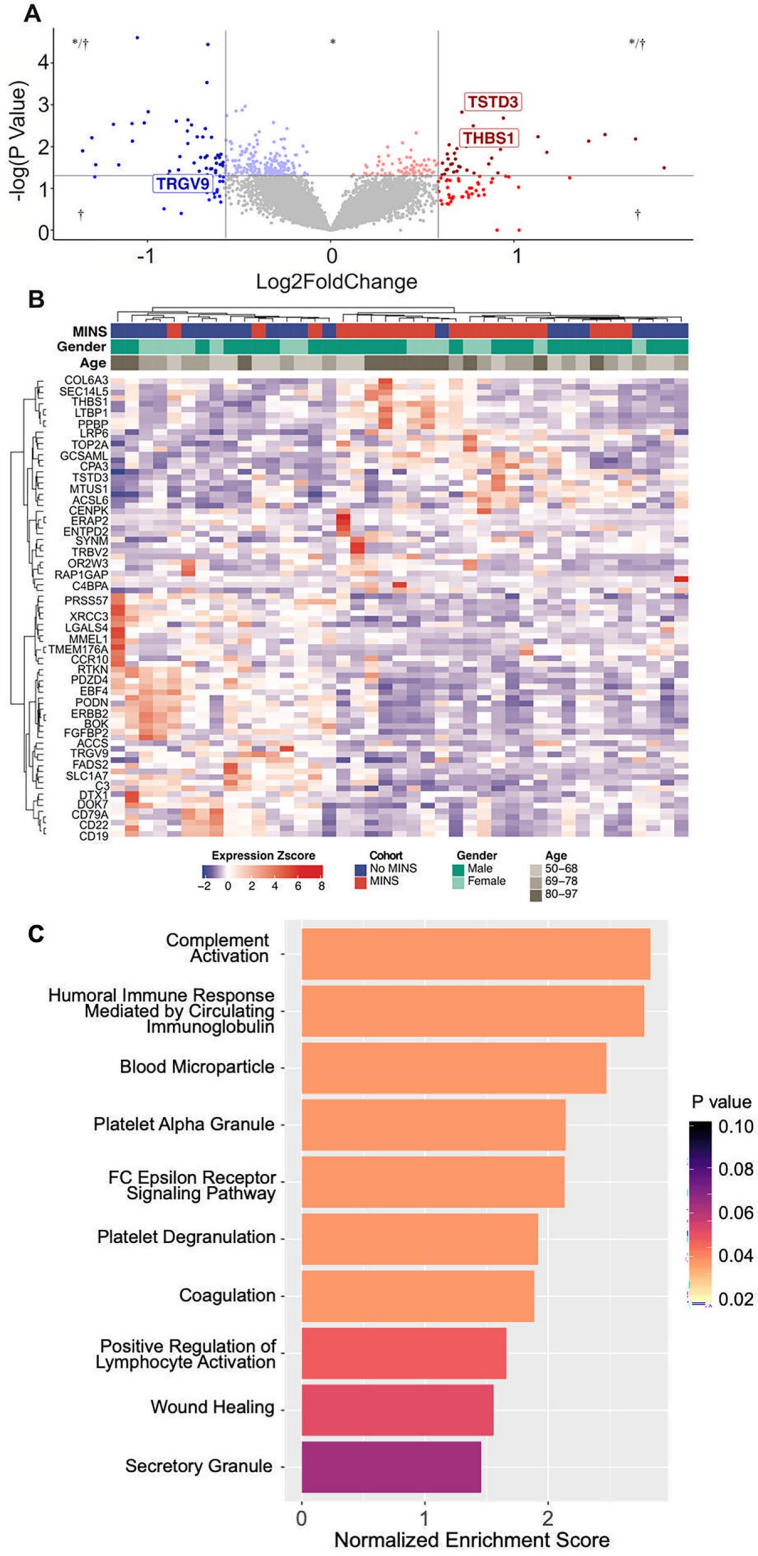
(**A**) Volcano plot highlighting differentially expressed genes in participants with MINS versus without MINS (n = 41), with genes of interest highlighted; * nominal p<0.01, †log2fe > 0.5. (**B**) Heat map of differentially expressed genes clustered by MINS status; (**C**) Selection of Enriched Gene Sets in patients with MINS, with Normalized Enrichment Scores for each GO term. Adjusted p values were used for the gene set enrichment analysis for all GO terms.

In an exploratory analysis, transcriptome profiling analysis identified 353 genes differentially expressed with a nominal p < 0.05. Using a stricter cut-off of a log2 fold change of > 0.5 and a nominal p < 0.05, 81 genes were differentially expressed (Fig. 2A, Supplemental Table 4). Figure 2B shows results of the hierarchical clustering. Gene set enrichment analysis of all 13,211 genes revealed global transcriptional shifts between participants with and without MINS (Fig. 2C).

To confirm our findings in a cohort without the potential for undiagnosed myocardial injury prior to surgery, we performed differential expression analysis in a smaller group of PACE participants with normal pre-operative troponin levels. A subset of 5 participants with MINS and normal pre-operative troponin values (mean age 67.4 ± 4.6 years, 1 female, peak post-operative troponin mean 2.98 ± 5.84 ng/mL, median 0.28 [0.09–1.06]) were matched 1:1 with 5 participants with normal pre-operative troponin values without MINS (mean age 68.8 ± 4.5 years, 2 females, peak post-operative troponin mean 0.012 ± 0.001 ng/mL, median 0.012 [0.012–0.013]). A total of 28 genes were differentially expressed with a log2 fold change > 0.5 and an adjusted p-value of < 0.01. Differentially expressed genes clustered participants by MINS status, as shown in Supplemental Fig. 1.

**Figure 3:**
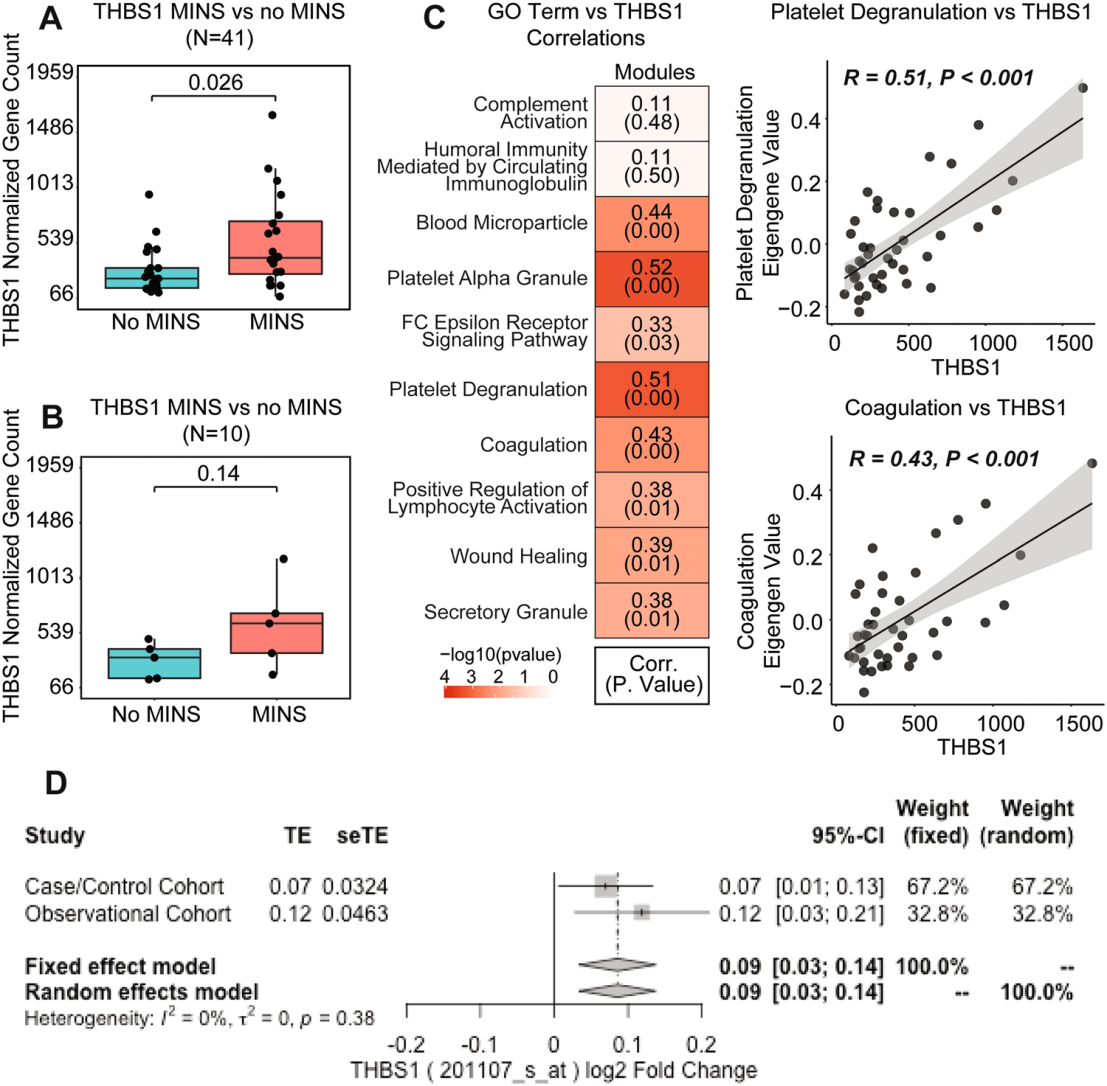
(**A**, **B**) Thrombospondin 1 (THBS1) gene expression in participants with MINS (Red) versus no MINS (Blue) in the overall cohort (**A**), and in a sensitivity analysis with negative pre-operative troponin values (**B**). (**C**) Correlations between THBS1 expression and relevant GO genesets were observed in 8 pathways; scatterplots of THBS1 and GO platelet degranulation and coagulation genesets are shown in greater detail. (**D**) Pooled differential expression analyses of THBS1 in non-surgical patients with and without cardiovascular events over long-term follow-up in observational and case–control cohorts from the CATHGEN database (observational cohort log2-fold change 0.12, 95% CI 0.03–0.21, p = 0.049; aOR 4.93; 95% CI 1.41–17.22; p = 0.012; case–control cohort log2-fold change 0.07, 95% CI 0.01–0.13, p = 0.143; aOR 2.03; 95% CI 1.06–3.87; p = 0.032).

To strengthen our exploratory differential expression analysis, we only investigated genes identified by multiple analyses comparing patients with MINS versus without MINS. Specifically, we looked at the overlap in differentially expressed genes found in (a) cohort of 20 MINS vs. 21 without MINS (n = 41); (b) a sensitivity analysis of 5 MINS vs. 5 without MINS, all with normal pre-operative troponin values (n = 10); and (c) a sensitivity analysis with MINS defined as a post-operative troponin > 0.1 ng/mL vs. no MINS (n = 32). Based on the intersection of these three analyses, we identified 3 genes of interest that were differentially expressed with a nominal p value < 0.05 and a log2 fold change of > 0.5 in participants with MINS (Supplemental Fig. 2). Two genes, *THBS1* (Thrombospondin 1) and *TSTD3* (Thiosulfate Sulfurtransferase Like Domain Containing 3 gene) were upregulated in MINS (Fig. 3A,B, Supplemental Fig. 3A,B). *TRGV9* (T Cell Receptor Gamma Variable 9) was consistently downregulated in participants with MINS (Supplemental Fig. 3C,D). Two gene sets were enriched in MINS in multiple analyses (adjusted p-values < 0.2): platelet alpha granule membrane and coagulation. Gene sets for ribosome biogenesis and cytosolic ribosomes were consistently downregulated in MINS. We observed correlations between *THBS1* expression and 8 pathways relevant to thrombosis defined by gene sets, including platelet degranulation, platelet alpha granules, coagulation, complement activation and humoral immune responses (Fig. 3C).

**Figure 4:**
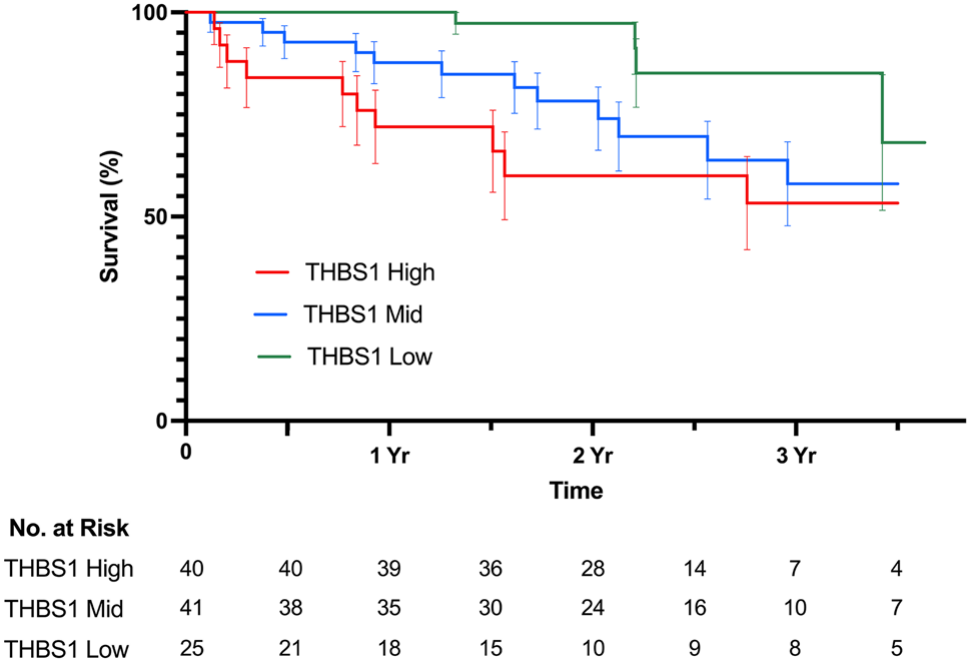
Kaplan Meier curves depicting freedom from death after lower extremity revascularization by tertile of pre-operative THBS1 expression.

We evaluated the relationship between pre-operative *THBS1* expression and long-term clinical outcomes in our cohort of PAD patients undergoing lower extremity revascularization. Kaplan Meier curves illustrate survival in patients by tertile of *THBS1* expression in Fig. 4 (log rank p = 0.015). Individuals with the highest tertile of pre-operative *THBS1* expression had an increased age- and sex-adjusted hazard of long-term mortality (aHR 3.71, 95% CI 1.12–12.28, p = 0.027, Supplemental Fig. 4) and the composite endpoint of death or MI (aHR 2.82, 95% CI 1.01–7.91) compared to the lowest tertile of *THBS1* expression.

Finally, we evaluated the expression of *THBS1* in RNA microarray data from two CATHGEN cohorts with long-term longitudinal follow-up for cardiovascular events. Higher *THBS1* mRNA levels (probe set 201107_s_at) were associated with increased risk for incident cardiovascular events after adjusting for age, sex, and race across cohorts (meta-analysis OR 2.67; 95% CI 1.19–6.00; p = 0.017; and meta-analysis log2-fold change, 0.086; 95% CI 0.034–0.148; p = 0.016) and was consistent within each cohort (Fig. 3D). In contrast, *TSTD3* was not differentially expressed in patients with and without cardiovascular events in the CATHGEN cohorts (data not shown). Microarrays did not include probes for *TRGV9*, and as a consequence, differential expression of this gene could not be evaluated in the CATHGEN database.

## Discussion

In a prospective cohort of patients with PAD undergoing surgical or endovascular lower extremity revascularization with longitudinal follow-up, MINS occurred in 23.5% of patients and was independently associated with long-term cardiovascular events. Patients with MINS were more likely to have coronary artery disease, heart failure, low hemoglobin, an increased post-operative creatinine, or a prolonged operative time than those without MINS. Patients who developed MINS were also more likely to present with evidence of critical limb ischemia, including ulceration and gangrene. In multivariable models, a pre-operative diagnosis of CAD was an independent predictor of MINS. In an exploratory analysis of pre-operative whole blood transcription profiles that did not account for multiple testing, the platelet alpha granule gene set and thrombospondin 1 genes were upregulated in patients who subsequently developed MINS. Pre-operative Thrombospondin 1 expression was also associated with survival after lower extremity revascularization and long-term cardiovascular events when evaluated in independent cohorts of non-surgical patients with cardiovascular disease. To our knowledge, this represents the first analysis of whole blood transcriptome profiles prior to surgery among surgical patients with and without post-operative myocardial injury, and the first study to explore pre-operative mechanisms of MINS.

In the current study, the incidence of MINS was similar to that reported in a meta-analysis of high quality, prospective studies with systematic post-operative troponin surveillance^5^. Although the association between MINS and 30-day mortality is well established, few studies have prospectively followed patients beyond 1 year^5,27^. We observed a strong association between MINS and MACE over long-term follow up in patients undergoing lower extremity revascularization.

The relationship between pre-operative expression of platelet and coagulation related genes in whole blood of patients prior to MINS is a novel finding that provides insights into a potential mechanism for post-operative ischemic events. A set of genes related to platelet alpha granules were upregulated in patients who subsequently developed MINS. Contents of platelet alpha granules include platelet-derived growth factors, platelet factor 4 and other contents that may pre-dispose to thrombosis, including thrombospondin-1, fibronectin, factor V, and von Willebrand factor. Thrombospondin 1 is of particular interest, since this gene was consistently upregulated in whole blood transcriptional analyses in patients with MINS. A subunit of a disulfide-linked homotrimeric protein, Thrombospondin 1 is an adhesive glycoprotein that can bind fibrinogen, fibronectin, laminin, type V collagen and integrins alpha-V/beta-1. The identification of upregulated coagulation-related genes may also provide a mechanistic foundation that substantiates the results of a large randomized clinical trial that suggests antithrombotic therapy may confer long-term benefit in patients with MINS^28^. Furthermore, since transcriptional differences in patients with and without MINS were observed prior to surgery, thrombospondin-1 may also serve as a potential pre-operative biomarker to identify patients at the highest risk for post-operative ischemic events. Thrombospondin was also associated with long-term cardiovascular events in an independent cohort of non-surgical patients in this and prior analyses^12^. Further studies are necessary to determine whether the risk of MINS can be modulated with pre-operative therapy in select subgroups of at-risk patients identified based on transcriptional profiles or specific circulating biomarkers.

### Limitations

There are some notable limitations to the current analysis. First 22% of patients did not have troponin measured post-operatively and were excluded from the analysis. Since patients that did not undergo the protocol-mandated post-operative troponin measurement were also less likely to have had an independent clinical indication for troponin surveillance, this may introduce ascertainment bias and increase the risk profile of the cohort for analysis. Second, there was significant heterogeneity in the surgical approach. Some participants underwent open surgical revascularization while others underwent endovascular revascularization by the surgical team. While hemodynamic and inflammatory effects differ by surgical approach, there was no difference in the incidence of MINS between patients who underwent open or endovascular repair. Moreover, this cohort is representative of contemporary approaches to the operative management of PAD by vascular surgery. Third, although robust phenotyping was performed, pre-operative whole blood RNA was not collected for all participants. Fourth, a nominal p-value < 0.05 was used to identify differentially expressed genes in our exploratory analysis and was not adjusted for multiple testing. This may overestimate the significance of the observed associations, and consequently, the findings should be considered hypothesis generating and require validation in future studies. However, the analysis was strengthened by requiring potential genes of interest to be differentially expressed in multiple analyses comparing patients with versus without MINS, and we provide clinical validation using the Duke CATHGEN database. Nonetheless, the use of nominal p-values remains an important limitation of the differential gene expression analysis. Fifth, while all data were prospectively collected, we cannot exclude unmeasured confounders. Long-term follow up and event capture was robust, with study telephone calls twice annually, clinical visits, and queries of death databases. Despite these measures, under-reporting of non-fatal events cannot be excluded. Relatively few participants had follow-up data available beyond 2 years, limiting the confidence of long-term survival estimates.

## Conclusions

Myocardial injury after non-cardiac surgery is common after lower extremity revascularization for PAD and was independently associated with major adverse cardiovascular and limb events at long-term follow up. Pre-operative transcriptional profiles identified that coagulation and platelet alpha-granule gene sets, and Thrombospondin 1, an adhesive glycoprotein found in platelets, were differentially overexpressed in patients with MINS. The relationship between pre-operative pathways related to platelet activity and MINS is a novel finding that provides new insights into potential mechanisms and therapeutic targets for post-procedural myocardial injury.

## Supplementary Information

Supplementary Information.

## References

[1] Weiser TG, Haynes AB, Molina G (2015). Estimate of the global volume of surgery in 2012: An assessment supporting improved health outcomes. Lancet.

[2] Smilowitz NR, Berger JS (2020). Perioperative cardiovascular risk assessment and management for noncardiac surgery: A review. JAMA.

[3] Smilowitz NR, Gupta N, Ramakrishna H, Guo Y, Berger JS, Bangalore S (2017). Perioperative major adverse cardiovascular and cerebrovascular events associated with noncardiac surgery. JAMA Cardiol..

[4] Ruetzler K, Smilowitz NR, Berger JS (2021). Diagnosis and management of patients with myocardial injury after noncardiac surgery: A scientific statement from the american heart association. Circulation.

[5] Smilowitz NR, Redel-Traub G, Hausvater A (2019). Myocardial injury after noncardiac surgery: A systematic review and meta-analysis. Cardiol. Rev..

[6] Devereaux PJ, Biccard BM, Writing Committee for the VSI (2017). Association of postoperative high-sensitivity troponin levels with myocardial injury and 30-day mortality among patients undergoing noncardiac surgery. JAMA.

[7] Thygesen K, Alpert JS, Jaffe AS (2018). Fourth universal definition of myocardial infarction (2018). Circulation.

[8] Biccard BM, Scott DJA, Chan MTV (2017). Myocardial injury after noncardiac surgery (MINS) in vascular surgical patients: A prospective observational cohort study. Ann. Surg..

[9] Horwitz PA, Tsai EJ, Putt ME (2004). Detection of cardiac allograft rejection and response to immunosuppressive therapy with peripheral blood gene expression. Circulation.

[10] Wingrove JA, Daniels SE, Sehnert AJ (2008). Correlation of peripheral-blood gene expression with the extent of coronary artery stenosis. Circ. Cardiovasc. Genet..

[11] Sinnaeve PR, Donahue MP, Grass P (2009). Gene expression patterns in peripheral blood correlate with the extent of coronary artery disease. PLoS ONE.

[12] Voora D, Cyr D, Lucas J (2013). Aspirin exposure reveals novel genes associated with platelet function and cardiovascular events. J. Am. Coll. Cardiol..

[13] Dann R, Hadi T, Montenont E (2018). Platelet-derived MRP-14 induces monocyte activation in patients with symptomatic peripheral artery disease. J. Am. Coll. Cardiol..

[14] Barrett TJ, Schlegel M, Zhou F (2019). Platelet regulation of myeloid suppressor of cytokine signaling 3 accelerates atherosclerosis. Sci. Transl. Med..

[15] Botto F, Alonso-Coello P, Chan MT (2014). Myocardial injury after noncardiac surgery: A large, international, prospective cohort study establishing diagnostic criteria, characteristics, predictors, and 30-day outcomes. Anesthesiology.

[16] Menard MT, Farber A, Assmann SF (2016). Design and rationale of the best endovascular versus best surgical therapy for patients with critical limb ischemia (BEST-CLI) trial. J. Am. Heart Assoc..

[17] Newman JD, Cornwell MG, Zhou H (2021). Gene expression signature in patients with symptomatic peripheral artery disease. Arterioscler. Thromb. Vasc. Biol..

[18] *Analysis pipelines for sequencing data.* 2020. Accessed 2020/03/03/16:54:25. https://github.com/igordot/sns

[19] Liao Y, Smyth GK, Shi W (2014). featureCounts: An efficient general purpose program for assigning sequence reads to genomic features. Bioinformatics.

[20] Dobin A, Davis CA, Schlesinger F (2013). STAR: Ultrafast universal RNA-seq aligner. Bioinformatics.

[21] Bolger AM, Lohse M, Usadel B (2014). Trimmomatic: A flexible trimmer for Illumina sequence data. Bioinformatics.

[22] Wingett SW, Andrews S (2018). FastQ Screen: A tool for multi-genome mapping and quality control. F1000Research..

[23] Alsaileek A, Nasim M, Aljizeeri A, Alharthi M, Al-Mallah MH (2014). The role of delayed contrast-enhanced cardiac magnetic resonance in differentiating myocarditis from myocardial infarction. Eur. Heart J. Suppl..

[24] Yu G, Wang L-G, Han Y, He Q-Y (2012). clusterProfiler: An R package for comparing biological themes among gene clusters. OMICS J. Integr. Biol..

[25] *msigdbr: MSigDB Gene Sets for Multiple Organisms in a Tidy Data Format*. Version 7.0.1. 2019. Accessed 2020/03/03/16:56:22. https://CRAN.R-project.org/package=msigdbr

[26] Gu Z, Eils R, Schlesner M (2016). Complex heatmaps reveal patterns and correlations in multidimensional genomic data. Bioinformatics.

[27] Oberweis BS, Smilowitz NR, Nukala S (2015). Relation of perioperative elevation of troponin to long-term mortality after orthopedic surgery. Am. J. Cardiol..

[28] Devereaux PJ, Duceppe E, Guyatt G (2018). Dabigatran in patients with myocardial injury after non-cardiac surgery (MANAGE): An international, randomised, placebo-controlled trial. Lancet.

