# Supplementary material for: Risk factors, transcriptomics, and outcomes of myocardial injury following lower extremity revascularization
Source: Sci Rep. 2022 Apr 25;12:6718. doi: 10.1038/s41598-022-10241-9 (PMC9038775; doi:10.1038/s41598-022-10241-9)

SUPPLEMENTAL MATERIALS

Supplemental Table 1. Characteristics of patients with and without post-operative measurement of troponin.

	All<br>(n=289)	Post-Op Troponin Measured<br>(n=226)	No Post-Operative Troponin<br>(n=63)	P-value
Age, median [IQR]	73.0 [65.0-80.0]	73 [66.0 - 81.0]	71.0 [63.5-76.0]	0.048
Female Sex	95 (32.9%)	79 (35.0%)	16 (25.4%)	0.20
Race				0.43
White	177 (61.2%)	141 (62.4%)	36 (57.1%)	
African American	66 (22.8%)	48 (21.2%)	18 (28.6%)	
Asian	5 (1.7%)	5 (2.2%)	0 (0.0%)	
Other	41 (14.2%)	32 (14.2%)	9 (14.3%)	
Ethnicity	55 (19.0%)	40 (17.7%)	15 (23.8%)	0.36
BMI, median [IQR]	26.3 [23.0-29.7]	26.1 [23.0-29.7]	27.2 [23.1-29.6]	0.89
Smoker				0.33
Current	49 (17.0%)	37 (16.4%)	12 (19.0%)	
Former	168 (58.1%)	132 (58.4%)	36 (57.1%)	
Never	72 (24.9%)	57 (25.2%)	15 (23.8%)	
Hypertension	254 (87.9%)	202 (89.4%)	52 (82.5%)	0.21
Hyperlipidemia	215 (74.4%)	163 (72.1%)	52 (82.5%)	0.13
Diabetes Mellitus	153 (52.9%)	118 (52.2%)	35 (55.6%)	0.74
Coronary Artery Disease	158 (54.7%)	122 (54.0%)	36 (57.1%)	0.76
Prior Myocardial Infarction	75 (26.0%)	64 (28.3%)	11 (17.5%)	0.12
Heart Failure	55 (19.0%)	47 (20.8%)	8 (12.7%)	0.21
Prior Stroke / TIA	52 (18.0%)	41 (18.1%)	11 (17.5%)	0.90
Obstructive Sleep Apnea	12 (4.2%)	8 (3.5%)	4 (6.3%)	0.53
Chronic Obstructive Pulmonary Disease	54 (18.7%)	39 (17.3%)	15 (23.8%)	0.32
Malignancy	64 (22.1%)	51 (22.6%)	13 (20.6%)	0.88
**Peripheral Artery Disease Classification**				0.22
Stable Peripheral Artery Disease	52 (18.0%)	36 (15.9%)	16 (25.4%)	
Critical Limb Ischemia	233 (80.6%)	187 (82.7%)	46 (73.0%)	
Femoral or Popliteal Aneurysm	4 (1.4%)	3 (1.3%)	1 (1.6%)	

Supplemental Table 2. Pre-operative predictors of myocardial injury after lower extremity revascularization.

Characteristic	aOR (95% CI)
Age	1.01 (0.97-1.05)
Male Sex	0.79 (0.38-1.64)
Race	
White	Reference
African American	0.51 (0.19-1.33)
Asian	1.99 (0.30-13.35)
Other	1.04 (0.19-5.76)
Hispanic Ethnicity	0.46 (0.09-2.26)
BMI (kg/m2)	0.96 (0.90-1.03)
Coronary Artery Disease	2.50 (1.13-5.52)*
Heart failure	1.58 (0.72-3.50)
Creatinine (mg/dL)	1.19 (0.98-1.44)
Hemoglobin (g/dL)	0.94 (0.78-1.13)

Multivariable logistic regression model includes demographics, pre-operative creatinine, and all other baseline (pre-operative) covariates with fewer than 5 missing values and with univariate p-values <0.1.
* $p<0.05$

Supplemental Table 3. Clinical outcomes over long-term follow up after lower extremity revascularization in a sensitivity analysis of 200 patients.

	**All** (n=200)	**No MINS** (n=163)	**MINS** (n=37)	**Unadjusted HR (95% CI)**	**Adjusted HR* (95% CI)**
MACLE	95 (47.5%)	71 (43.6%)	24 (64.9%)	2.15 (1.35-3.43)	2.20 (1.28-3.78)
MACE	49 (24.5%)	32 (19.6%)	17 (45.9%)	3.75 (2.07-6.81)	3.22 (1.59-6.52)
MALE	61 (30.5%)	49 (30.1%)	12 (32.4%)	1.23 (0.65-2.31)	1.63 (0.80-3.34)
Death	38 (19.0%)	26 (16.0%)	12 (32.4%)	2.74 (1.37-5.48)	1.70 (0.75-3.85)
Myocardial Infarction	20 (10%)	8 (4.9%)	12 (32.4%)	9.65 (3.90-23.90)	13.83 (4.49-42.63)
Death or MI	47 (23.5%)	30 (18.4%)	17 (45.9%)	4.18 (2.28-7.64)	3.75 (1.82-7.75)
Stroke	7 (3.5%)	6 (3.7%)	1 (2.7%)	1.03 (0.12-8.88)	0.54 (0.05-6.29)
Amputation	51 (25.5%)	35 (21.5%)	16 (43.2%)	2.33 (1.29-4.22)	3.01 (1.47-6.18)
Death or Amputation	79 (39.5%)	56 (34.4%)	23 (62.2%)	2.41 (1.47-3.93)	2.34 (1.31-4.19)

MACE: Major adverse cardiovascular events
MALE: Major adverse limb events
MACLE: Major adverse cardiovascular and limb events

* Cox proportional hazard models adjusted for age, sex, race/ethnicity, coronary artery disease, heart failure, baseline serum creatinine, and the surgical approach to revascularization.

Supplemental Table 4. Differentially expressed transcripts with p-value <0.05 and log2 fold change > 0.5 (n=81).

Gene Identifier	baseMean	log2FoldChange	pvalue
HLA-V	265.551003	1.820569089	0.03217864
RAP1GAP	126.0192	1.664331375	0.00657578
IGHV3-30	68.3912701	1.497752606	0.00517113
C4BPA	50.4393007	1.408284699	0.00738562
IGHV3-49	35.3638216	1.179854416	0.01374983
CENPK	49.9005134	1.131742414	0.00580448
MMRN1	45.7307141	0.94262235	0.00207844
MTUS1	22.9145487	0.927260849	0.01170081
IGLV3-1	147.179529	0.91344806	0.04264994
ENTPD2	49.5114121	0.878280251	0.01889305
AC005332.2	22.163111	0.862315773	0.02601242
TRBV2	50.8284242	0.840061785	0.00775847
OR2W3	187.10971	0.782500108	0.04297119
COL6A3	23.6273626	0.778533347	0.00319163
SEC14L5	31.9833764	0.743025644	0.03652217
ELOVL7	31.981234	0.740444518	0.00799007
ERAP2	779.99748	0.739545753	0.00981431
TOP2A	31.3906616	0.723286839	0.00542552
TSTD3	21.1593259	0.715597422	0.00149665
GCSAML	22.0576678	0.706322379	0.03065666
PROS1	27.4658967	0.698767332	0.02966588
TRAV13-2	27.7747806	0.689508139	0.03865816
THBS1	422.000875	0.688944338	0.01117862
PPBP	1588.65356	0.680868734	0.01980596
CPA3	75.1364952	0.675308134	0.01454342
AL731559.1	37.6674833	0.67175197	0.04046541
RAB6B	41.7956687	0.663486131	0.02581122
ACSL6	58.1243387	0.655468808	0.02658086
NCAPG2	88.4914072	0.646959689	0.00904898
LTBP1	73.0995534	0.640903777	0.01972019
AKAP12	49.9428971	0.637905699	0.03488571
SYNM	37.2256486	0.636569863	0.01504645
HEMGN	145.216757	0.631197507	0.03592959
TENT5C	1880.70948	0.62241295	0.04551361
LRP6	16.9735227	0.610505903	0.0247253
GABPB1-AS1	332.274627	0.610146956	0.02495335
NFXL1	155.679796	0.607843807	0.04731124
C3	63.6819095	-0.587602232	0.02884668
PDZD4	706.478782	-0.588596636	0.02409918
CHRNE	69.9569249	-0.598934298	0.01531951
TRGV10	28.294877	-0.603450671	0.02682526
AC017104.1	16.7346619	-0.607078833	0.01828401
RTN4R	29.6478374	-0.616502674	0.01610217
ARVCF	113.985368	-0.623516206	0.02498597
CCR10	27.5931415	-0.627875176	0.03724809

CD22	749.056497	-0.628192395	0.01880368
TRGV9	29.6883809	-0.628200315	0.04226222
ERBB2	225.041194	-0.649401452	0.03382256
CD72	218.230283	-0.651194788	0.00599448
PCDH1	37.8081864	-0.656607936	0.0341843
XRCC3	107.825537	-0.670776154	3.59E-05
PODN	21.5477457	-0.673400068	0.04756345
EBF4	44.8409725	-0.675602591	0.01664556
RUFY4	59.0307721	-0.675981234	0.00029352
VPREB3	67.6192447	-0.679209074	0.03299258
ACCS	344.039554	-0.684584302	0.00371803
AL031846.1	25.1153681	-0.685651794	0.01831746
LAMB2	69.4548397	-0.698134711	0.00590127
FGFBP2	903.890402	-0.710089856	0.01812109
SPON2	1590.0233	-0.717740114	0.03867598
DTX1	109.027465	-0.728583132	0.00584597
BICDL2	68.0369705	-0.745534957	0.0246109
CD19	271.650754	-0.759641126	0.00303354
AC091729.3	17.6202347	-0.77955339	0.0022885
RTKN	21.2905155	-0.78032387	0.00844023
CD79A	1166.40116	-0.78257088	0.00424687
FADS2	273.60061	-0.823510171	0.01735876
SLC1A7	72.9145523	-0.831086529	0.0360011
DOK7	19.1658896	-0.842842087	0.00245524
OTOF	28.5601326	-0.879906564	0.03879577
LAIR2	41.8344178	-0.887176667	0.0473117
COPZ2	25.6326022	-0.996729885	0.00146431
AC123912.4	47.7288776	-1.017760072	0.00270492
MMEL1	17.5979995	-1.055787625	2.47E-05
PRSS57	37.2170298	-1.082702849	0.00740556
LGALS4	27.7609929	-1.085204883	0.00281495
TMEM176B	901.20349	-1.15742751	0.02743491
ZNF683	239.245593	-1.185880375	0.00293066
BOK	69.6742283	-1.282597659	0.02719164
TMPRSS9	39.4164692	-1.303812748	0.00614427
TMEM176A	434.963127	-1.354946036	0.0126953

Supplemental Figure 1. Differentially expressed genes with participants clustered by MINS status in a subset of 5 participants with MINS and normal pre-operative troponin values matched to 5 participants with normal pre-operative troponin values without MINS (n=10)

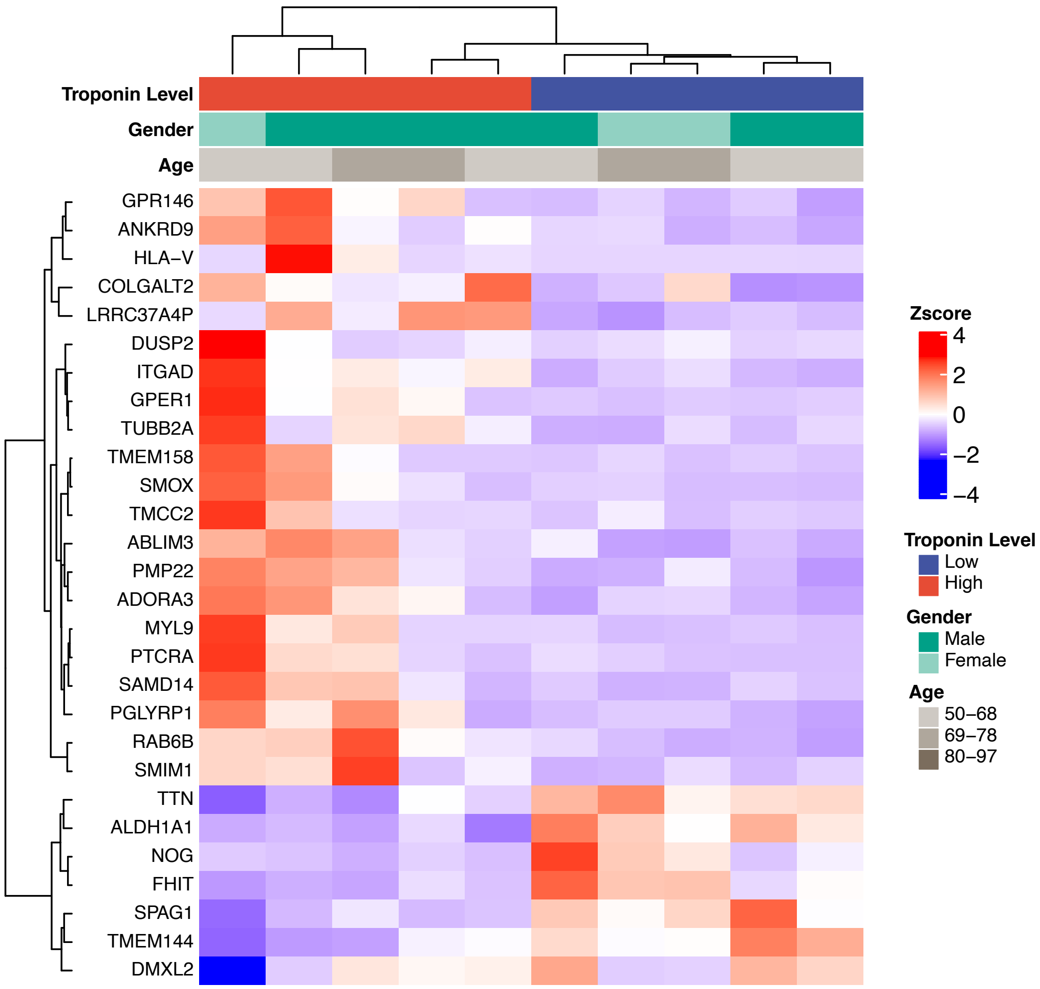

Supplemental Figure 2: Venn diagram of differentially expressed genes in 3 analyses of whole blood from patients with MINS versus without MINS: (a) cohort of 20 MINS vs. 21 without MINS; (b) a sensitivity analysis of 5 MINS vs. 5 without MINS, all with normal pre-operative troponin values; and (c) a sensitivity analysis with MINS defined as a post-operative troponin >0.1 ng/mL vs. no MINS. Genes of interest were identified if differential expression in patients with MINS was observed in all 3 analyses.

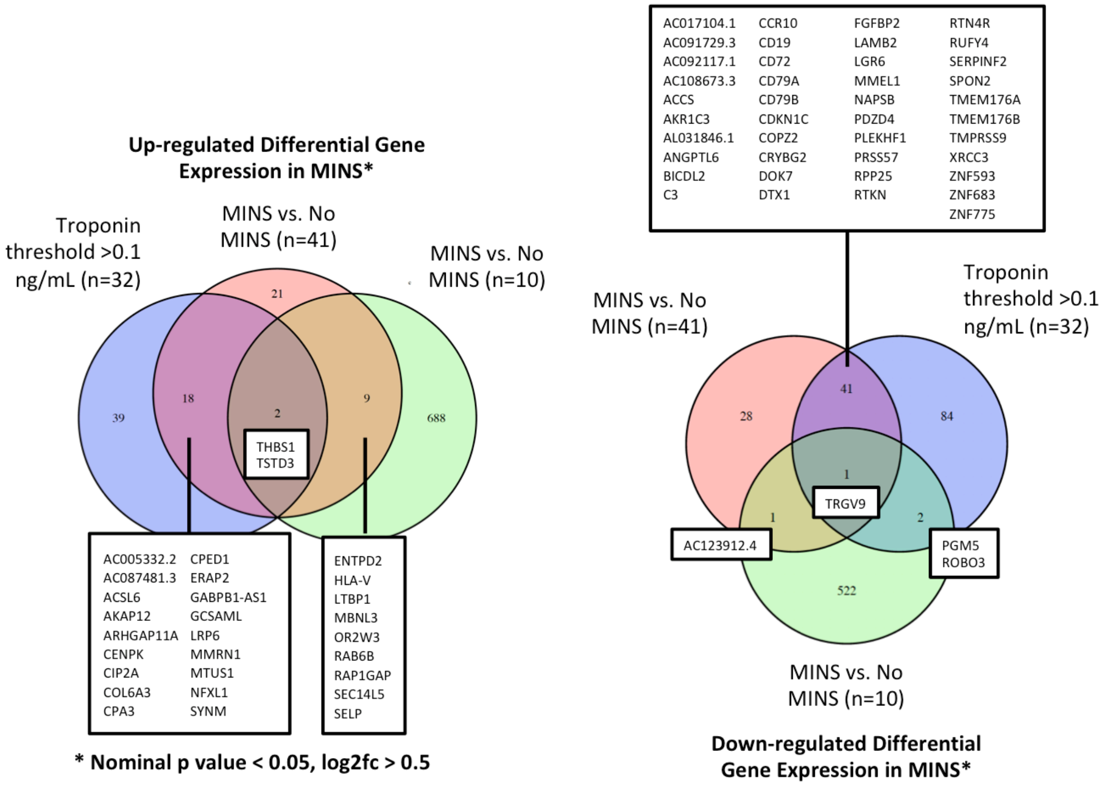

Supplemental Figure 3: Thiosulfate Sulfurtransferase Like Domain Containing 3 (TSTD3) (**Panels A & B**) and T Cell Receptor Gamma Variable 9 (TRGV9) (**Panels C & D**) gene expression in participants with MINS (Red) versus no MINS (Blue). **Panels A, C** show data from the overall cohort (n=41; 20 MINS vs. 21 without MINS) while **Panels B, D** show data from a sensitivity analysis of matched participants with MINS versus no MINS who had normal pre-operative troponin values (N=10; 5 MINS vs. 5 without MINS).

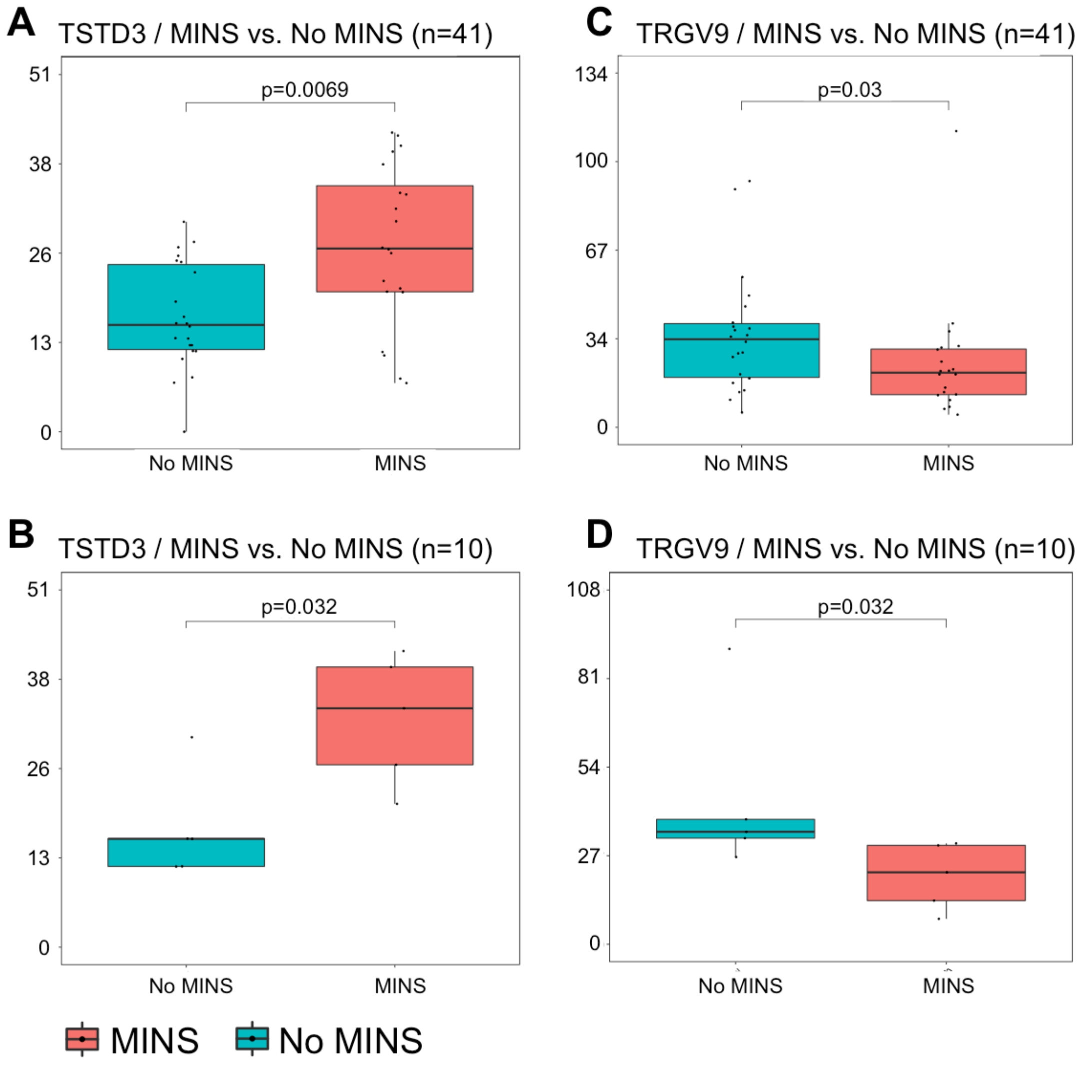

Supplemental Figure 4. Hazard of long-term mortality associated with tertiles of THBS1 expression, after adjustment for age and sex, in patients undergoing lower extremity revascularization.

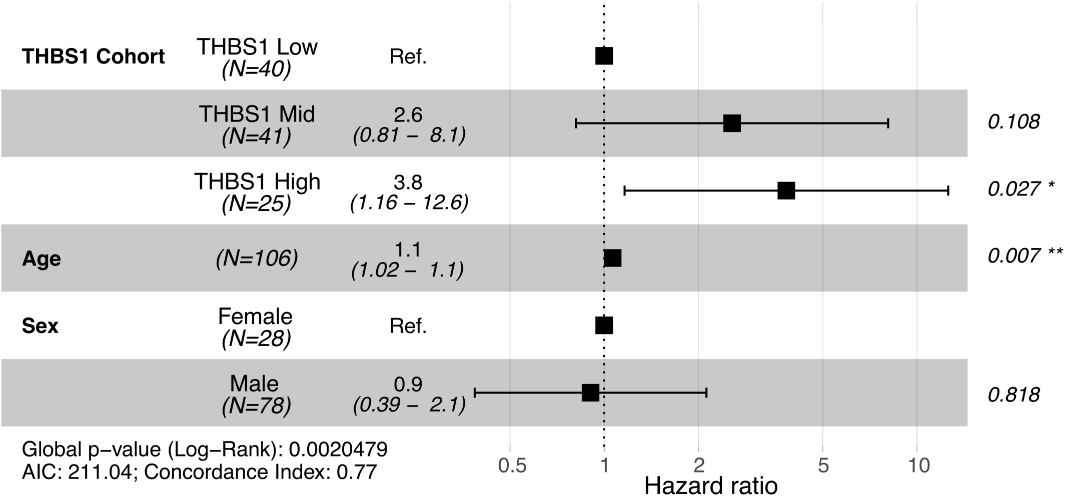

Supplement: Supplementary file 1 — Supplementary Information. [file 41598_2022_10241_MOESM1_ESM.pdf]
